# An Increase in Same-day Discharge After Total Joint Arthroplasty During the COVID-19 Pandemic Does Not Influence Patient Outcomes: A Retrospective Cohort Analysis

**DOI:** 10.1016/j.artd.2023.101115

**Published:** 2023-02-08

**Authors:** Brook A. Mitchell, Liam M. Cleary, Linsen T. Samuel, Benjamin R. Coobs, Miles A. Thomas, Stephen C. Martinkovich, Joseph T. Moskal

**Affiliations:** a Virginia Tech Carilion School of Medicine, Carilion Clinic, Roanoke, VA, USA; b Department of Orthopaedic Surgery, Institute for Orthopaedics & Neurosciences, Carilion Clinic, Roanoke, VA, USA; c Department of Orthopaedic Surgery, Adult Reconstruction, Allegheny Health Network, Pittsburgh, PA, USA

**Keywords:** Total hip arthroplasty (THA), Total knee arthroplasty (TKA), Length of stay, Same day discharge, Outpatient

## Abstract

**Background:**

The coronavirus disease 2019 (COVID-19) pandemic caused major transitions in total joint arthroplasty (TJA), notably with the increased utilization of same-day discharge (SDD) pathways. This study assessed the effect of accelerated discharge pathways following the resumption of elective cases during the COVID-19 pandemic on SDD rates, adverse events, and characteristics associated with successful SDD following total hip and total knee arthroplasty.

**Methods:**

This retrospective study split patients into cohorts: TJA prior to COVID-19 (pre-COVID, July 2019-December 2019) and TJA following the resumption of elective surgeries (post-COVID, July 2020-December 2020). Patient characteristics such as age, sex, body mass index, American Society of Anesthesiologists score, and pertinent comorbidities were analyzed, and length of stay, 30-day emergency department (ED) visit rates, readmissions, and reoperations were compared.

**Results:**

A total of 1333 patients met inclusion criteria that were divided into pre-COVID (692) and post-COVID (641) cohorts. The pre-COVID group had a median age of 69 years (interquartile range 63-76), and the post-COVID group had a median age of 68 years (interquartile range 61-75) (*P* = .024). SDD increased from 0.1% to 28.9% (*P* < .001), and length of stay decreased from 1.3 days to 0.89 days (*P* < .001). There was no change in 30-day ED visits, readmissions, or reoperations (*P* = .817, *P* = .470, and *P* = .643, respectively). There was no difference in ED visits, readmissions, or reoperations in SDD patients. The odds of SDD were associated with age (*P* < .001, odds ratio [OR] = 0.94), body mass index (*P* = .006, OR = 0.95), male sex (*P* < .001, OR = 1.83), and history of tobacco use (*P* < .001, OR = 1.87).

**Conclusions:**

At our institution, the COVID-19 pandemic accelerated the utilization of SDD pathways without increasing ED visits, readmissions, or reoperations.

## Introduction

The effects of the novel coronavirus disease 2019 (COVID-19) pandemic have been felt worldwide for the past 2 years. To support the sudden demand for personal protective equipment and ventilators necessary to care for those most directly affected by the pandemic, there was a temporary disruption to the provision of elective orthopaedic surgery services in the United States [[1], [2], [3], [4], [5]]. As our hospital infrastructure adapted, continued efforts to optimize care have catalyzed a shift towards more resource-efficient outpatient total joint arthroplasty (TJA) [6,7].

The removal of total knee arthroplasty (TKA) and total hip arthroplasty (THA) from the inpatient-only (IPO) list has allowed orthopaedic surgeons to integrate outpatient TJA, utilizing same-day discharge (SDD) to mitigate the inpatient burden of COVID-19 [8]. Even prior to the pandemic, SDD was gaining momentum with an increase in prevalence from 0.95% to 20.5% between 2011 and 2015 [9]. Low complication rates have been observed, with rates as low as 1.98% for both readmission and reoperation in large multicenter studies [10]. Furthermore, no statistical difference has been found in 90-day emergency department (ED) visits and 90-day hospital readmission rates between SDD and later discharge [11]. Several other large studies have also shown there is either no statistical difference in safety profiles of inpatient vs outpatient TJA or outpatient TJA was associated with less adverse events; however, conflicting studies have found higher rates of revision in outpatient TKA [[12], [13], [14], [15], [16], [17]].

Rapidly adopting safe SDD practices may serve to relieve the stress on the health-care system and allow patients to undergo procedures that have a profound impact on their quality of life [10,11,17,18]. The primary purpose of this investigation was to evaluate the safety of the rapid increase in SDD practices without specific patient selection criteria. Our hypothesis was that SDD practices would increase following resumption of elective TJA without increasing postoperative ED visits, readmissions, or reoperations. We aim to determine if there was a change in SDD rate, and if so, what that change was, surrounding the halting and resumption of elective TJA. We also aimed to determine the 30-day ED visit rate, 30-day reoperation rate, and 30-day readmission rate after SDD in TJA and any patient characteristics that may increase the likelihood of successful SDD.

## Material and methods

### Study design

**Figure 1:**
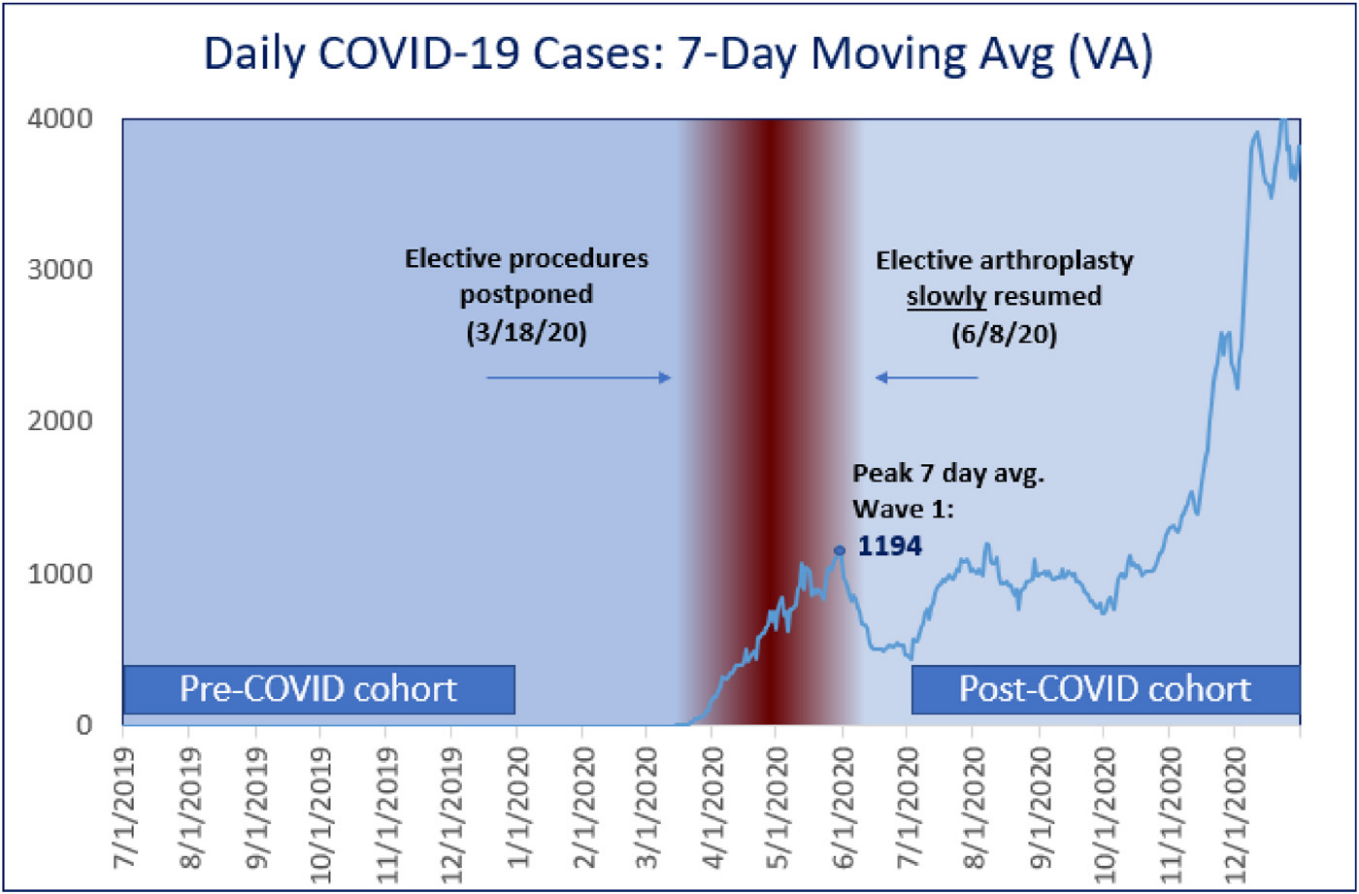
Timeframe of elective procedures postponement and resumption, and cohort timeframes, compared to the seven-day moving average of daily COVID-19 cases in the state of Virginia.

In response to the evolving COVID-19 pandemic and recommendations provided by the Centers for Medicare and Medicaid Services, no elective TJAs were performed at our center from March 18, 2020, through June 8, 2020 [19]. This facilitated the creation of 2 patient cohorts based on a discrete time period, described below. After obtaining approval from our institutional review board (IRB-21-1227), patient data were collected manually through retrospective chart review at a single tertiary care facility to identify eligible patients. Our inclusion criteria were patients who (1) underwent primary, elective THA (Current Procedural Terminology code 27130) or TKA (Current Procedural Terminology code 27447) (2) from July 2019 through December 2019 (pre-COVID) and from July 2020 through December 2020 (post-COVID). These timelines were chosen in concordance with other institution’s elective surgery pause in the state of Virginia (Fig. 1). Our exclusion criteria were patients who (1) underwent nonelective THA or TKA due to fracture/trauma, revision, or conversion; (2) had a bilateral procedure; and/or (3) underwent any alternative approach to the direct anterior (DA) one for THA. As the DA approach is the preferred one for THA for all surgeons at our institution, the rare use of alternative approaches is almost exclusively performed in atypical cases and was felt to be a confounding variable.

### Arthroplasty program changes

During the post-COVID study period, our center adopted a rapid recovery perioperative management care plan with an emphasis on virtual patient and caretaker education, aggressive physical therapy regimens, and optimized multimodal pain management. We prioritized earlier postoperative return of patient function through increased utilization of spinal over general anesthesia, decreased dosages of bupivacaine in spinal anesthesia, and the elimination of intrathecal narcotics. A standard approach to deep vein thrombosis (DVT) prophylaxis using aspirin was maintained throughout the study period for both cohorts.

Following resumption of elective arthroplasty, our institution utilized the Medically Necessary, Time-Sensitive Procedures Scoring system (MeNTS) to evaluate patients appropriate for elective TJA. This scoring system was developed to incorporate factors not regularly considered by surgeons, such as resource availability and the COVID-19 transmission risk of the patient [20]. Priority is given to short procedures performed outside of the viscera with estimated short length of stay, relatively little nonoperative treatment efficacy, and worsening of outcome over time without treatment. Contraindications to SDD were lack of an immediate support system in the discharge environment for 2 weeks and unanticipated events during admission.

### Surgical, demographic variables, and outcome measures

Patient demographics were collected, grouped, and analyzed, including age, body mass index (BMI), sex, and American Society of Anesthesiologists score (ASA). Data on pertinent past medical history and comorbidities were also collected and analyzed including diabetes mellitus, chronic obstructive pulmonary disease, obstructive sleep apnea, chronic kidney disease, history of tobacco use, stroke, pulmonary embolism (PE), or DVT. General LOS and SDD, defined as discharge of the patient on the same calendar day, were observed. Primary postoperative outcome measures including SDD rate and 30-day incidence of ED visit, hospital readmission, or reoperation were collected.

### Statistical analysis

All data were compiled in a Research Electronic Data Capture (REDCap) database and exported to R software (R Foundation for Statistical Computing, Vienna, Austria) for statistical analysis. Study data were collected and managed using REDCap electronic data-capture tools hosted at our institution [[21], [22], [23]]. Continuous variables were presented as mean and standard deviation for parametric variables and median and interquartile range (IQR), which is the range from the 25th to 75th percentiles, for nonparametric variables. Categorical variables were presented as frequency and percentage. A *P* value of < .05 was considered significant throughout the analysis. To compare differences in the pre-COVID and post-COVID cohorts, Student’s *t*-tests were used for continuous variables, and Chi-square tests were used for categorical variables. To determine factors that may alter the likelihood of SDD, univariate and multivariate binary logistic regression following stepwise model selection were used to analyze the post-COVID cohort only since the number of patients in the pre-COVID group discharged on the same day was not sufficient (1 patient). Results were reported as odds ratios (OR) and adjusted ORs.

## Results

There were 1380 patients identified who underwent primary THA or primary TKA during our timeframes. Of the 1380, 1333 patients met inclusion criteria (722 TKA, 611 THA). Of the 1333, 692 were in the pre-COVID group (296 THA, 396 TKA), and 641 were in the post-COVID group (315 THA, 326 TKA). The pre-COVID group had a median age of 69 (IQR 63-76) years, and the post-COVID group had a median age of 68 (IQR 61-75) years (*P* = .024). There were no other demographic differences when comparing overall the pre-COVID group to the post-COVID group. A 6.3% increase in THA volume relative to TKA volume was observed (*P* = .023).

SDD increased from the pre-COVID rate of 0.1% to the post-COVID rate of 28.9% (*P* < .001), with THA increasing from 0.3% to 33% (*P* < .001) and TKA increasing from 0% to 24.8% (*P* < .001). The mean LOS decreased from 1.3 (0.82) days for the pre-COVID group to 0.89 (0.95) days for the post-COVID group (*P* < .001). Only 1 patient (0.01%) was discharged on the same day in the pre-COVID cohort, so the post-COVID cohort was analyzed separately for differences in patients discharged on the same day (SDD) and those kept inpatient. Inpatients were older with a median age of 70 (IQR 63-77) years than SDD patients with a median age of 64 (IQR 58-70) years (*P* < .001). More SDD patients were male (55.1%) than inpatients (36.4%) (*P* < .001). ASA scores differed with a higher proportion of inpatients categorized as ASA 3 (50.4%) than SDD patients (30.8%) and a lower proportion of inpatients categorized as ASA 2 (47.8%) than SDD patients (67.6%) (*P* < .001). There were significantly more SDD patients with a history of tobacco use (23.8%) than inpatients (13.6%) (*P* = .002) and significantly less SDD patients with a history of stroke (1.6%) than inpatients (6.4%) (*P* = .022). Table 1 contains complete information of post-COVID patient demographics stratified by discharge timing.

**Table 1:** Post-COVID demographic information stratified by discharge timing.

Demographics	In-patient	Same-day discharge	*P*
n	456	185	
Age (median [IQR])	70.00 [63.00, 77.00]	64.00 [58.00, 70.00]	**<.001**
Male (%)	166 (36.4)	102 (55.1)	**<.001**
Body mass index (kg/m^2^) (median [IQR])	31.29 [28.25, 34.98]	30.57 [27.30, 34.84]	.195
American Society of Anesthesiologists score (%)			**<.001**
1	1 (0.2)	3 (1.6)	
2	218 (47.8)	125 (67.6)	
3	230 (50.4)	57 (30.8)	
4	7 (1.5)	0 (0.0)	
Packed red blood cells, mean (SD)	0.02 (0.28)	0.00 (0.00)	.250
No. of comorbidities, mean (SD)	1.33 (1.21)	1.17 (1.26)	.149
Comorbidities			
Diabetes (%)	99 (21.7)	35 (18.9)	.496
Stroke (%)	29 (6.4)	3 (1.6)	**.022**
Myocardial infarction (%)	33 (7.2)	7 (3.8)	.145
Deep vein thrombosis (%)	31 (6.8)	5 (2.7)	.064
Pulmonary embolism (%)	9 (2.0)	2 (1.1)	.651
Chronic kidney disease (%)	62 (13.6)	21 (11.4)	.524
Thyroid problem (%)	90 (19.7)	35 (18.9)	.899
Obstructive sleep apnea (%)	83 (18.2)	32 (17.3)	.875
Arrhythmia (%)	72 (15.8)	27 (14.6)	.796
Chronic obstructive pulmonary disease (%)	35 (7.7)	6 (3.2)	.057
Tobacco use history (%)	62 (13.6)	44 (23.8)	**.002**

IQR, interquartile range; SD, standard deviation.

Bold values: statistically significant.

There was no significant difference in 30-day ED visits between pre-COVID (7.7%) and post-COVID (7.2%) groups overall (*P* = .817) and between SDD (4.9%) and inpatient (8.1%) groups of the post-COVID cohort (*P* = .202). Similarly, there were no differences in instances of 30-day readmissions or reoperations. Table 2 outlines 30-day outcome measures comparing post-COVID patients based on discharge timing.

**Table 2:** Thirty-day outcomes of post-COVID cohort.

Variable	In-patient	Same-day discharge	*P*
Emergency department visit rate (%)	37 (8.1)	9 (4.9)	.202
Readmission rate (%)	20 (4.4)	3 (1.6)	.141
Reoperation rate (%)	5 (1.1)	0 (0.0)	.350

Following stratification by joint, there were significant differences in THA patient ASA scores with a higher proportion of patients having an ASA score of 3 in the post-COVID group (47.6%) than those in the pre-COVID group (37.8%) and a lower proportion of patients with an ASA score of 2 in the post-COVID group (50.8%) than those in the pre-COVID group (57.8%) (*P* = .031). No differences were found in ASA score proportions for patients who underwent TKA between the pre-COVID and post-COVID groups (*P* = .146). Post-COVID TKA patients had significantly lower prevalence of DVT history (4.6%) than pre-COVID patients (9.1%) (*P* = .028), and PE history (0.9%) than pre-COVID (4.5%). Table 3, Table 4 contain complete information of patient demographics and comorbidities stratified by joint and time period.

**Table 3:** Demographics and comorbidities of THA patients.

Variable	All TJA	All THA	Period (THA)		*P*
			Pre-COVID	Post-COVID	
n	1333	611	296	315	
Age (median [IQR])	69.00 [62.00, 76.00]	68.00 [61.00, 75.00]	68.00 [61.00, 75.00]	67.00 [60.00, 75.00]	.153
Sex					
Male (%)	534 (40.1)	252 (41.2)	118 (39.9)	134 (42.5)	.556
Female (%)	799 (59.9)	359 (58.8)	178 (60.1)	181 (57.5)	
Body mass index (kg/m^2^) (median [IQR])	31.68 [27.73, 35.31]	30.32 [26.81, 33.95]	30.11 [26.54, 34.27]	30.35 [27.56, 33.68]	.962
American Society of Anesthesiologists score (%)					**.031**
1	15 (1.1)	10 (1.6)	7 (2.4)	3 (1.0)	
2	705 (52.9)	331 (54.2)	171 (57.8)	160 (50.8)	
3	593 (44.5)	262 (42.9)	112 (37.8)	150 (47.6)	
4	20 (1.5)	8 (1.3)	6 (2.0)	2 (0.6)	
Packed red blood cells, mean (SD)	0.02 (0.22)	0.04 (0.31)	0.05 (0.31)	0.02 (0.30)	.251
No. of comorbidities, mean (SD)	1.31 (1.23)	1.20 (1.21)	1.19 (1.23)	1.22 (1.19)	.761
Comorbidities					
Diabetes (%)	293 (22.0)	126 (20.6)	61 (20.6)	65 (20.6)	1.000
Stroke (%)	61 (4.6)	32 (5.2)	17 (5.7)	15 (4.8)	.717
Myocardial infarction (%)	78 (5.9)	35 (5.7)	16 (5.4)	19 (6.0)	.874
Deep vein thrombosis (%)	95 (7.1)	44 (7.2)	23 (7.8)	21 (6.7)	.711
Pulmonary embolism (%)	35 (2.6)	14 (2.3)	6 (2.0)	8 (2.5)	.879
Chronic kidney disease (%)	167 (12.5)	74 (12.1)	38 (12.8)	36 (11.4)	.682
Obstructive sleep apnea (%)	259 (19.4)	113 (18.5)	52 (17.6)	61 (19.4)	.640
Arrhythmia (%)	202 (15.2)	87 (14.2)	42 (14.2)	45 (14.3)	1.000
Chronic obstructive pulmonary disease (%)	83 (6.2)	38 (6.2)	15 (5.1)	23 (7.3)	.329
Tobacco use history (%)	214 (16.1)	78 (12.8)	34 (11.5)	44 (14.0)	.425

IQR, interquartile range; SD, standard deviation.

Bold values: statistically significant.

**Table 4:** Demographics and comorbidities of TKA patients.

Variable	All TJA	All TKA	Period (TKA)		*P*
			Pre-COVID	Post-COVID	
n	1333	722	396	326	
Age (median [IQR])	69.00 [62.00, 76.00]	70.00 [63.00, 76.00]	70.00 [63.00, 77.00]	69.00 [62.00, 76.00]	.124
Sex					
Male (%)	534 (40.1)	282 (39.1)	148 (37.4)	134 (41.1)	.344
Female (%)	799 (59.9)	440 (60.9)	248 (62.6)	192 (58.9)	
Body mass index (kg/m^2^) (median [IQR])	31.68 [27.73, 35.31]	32.54 [28.72, 36.33]	32.97 [28.95, 37.07]	32.03 [28.37, 35.94]	.061
American Society of Anesthesiologists score (%)					.146
1	15 (1.1)	5 (0.7)	4 (1.0)	1 (0.3)	
2	705 (52.9)	374 (51.8)	191 (48.2)	183 (56.1)	
3	593 (44.5)	331 (45.8)	194 (49.0)	137 (42.0)	
4	20 (1.5)	12 (1.7)	7 (1.8)	5 (1.5)	
Packed red blood cells, mean (SD)	0.02 (0.22)	0.01 (0.11)	0.00 (0.00)	0.01 (0.16)	.119
No. of comorbidities, mean (SD)	1.31 (1.23)	1.39 (1.23)	1.43 (1.22)	1.34 (1.25)	.325
Comorbidities					
Diabetes (%)	293 (22.0)	167 (23.1)	98 (24.7)	69 (21.2)	.295
Stroke (%)	61 (4.6)	29 (4.0)	12 (3.0)	17 (5.2)	.195
Myocardial infarction (%)	78 (5.9)	43 (6.0)	22 (5.6)	21 (6.4)	.732
Deep vein thrombosis (%)	95 (7.1)	51 (7.1)	36 (9.1)	15 (4.6)	**.028**
Pulmonary embolism (%)	35 (2.6)	21 (2.9)	18 (4.5)	3 (0.9)	**.008**
Chronic kidney disease (%)	167 (12.5)	93 (12.9)	46 (11.6)	47 (14.4)	.314
Obstructive sleep apnea (%)	259 (19.4)	146 (20.2)	82 (20.7)	64 (19.6)	.791
Arrhythmia (%)	255 (19.1)	160 (22.2)	92 (23.2)	68 (20.9)	.500
Chronic obstructive pulmonary disease (%)	202 (15.2)	115 (15.9)	61 (15.4)	54 (16.6)	.748
Tobacco use history (%)	83 (6.2)	45 (6.2)	27 (6.8)	18 (5.5)	.574

IQR, interquartile range; SD, standard deviation.

Bold values: statistically significant.

Results of the univariate analysis found that there was a significant increase in odds of SDD if the patient was undergoing THA (OR 1.49 [1.06, 2.11]) compared to TKA (OR 0.67 [0.94, 0.47]) (Table 5). Patients were at higher odds of SDD if they were male (OR 2.15 [3.04, 1.52], *P* < .001) or if they smoke (OR 1.98 [3.05, 1.29], *P* = .002) and were at lower odds of SDD if they were older in age (OR 0.94 [0.96, 0.92], *P* < .001) or had a past medical history significant for stroke (OR 0.24 [0.69, 0.06], *P* = .02), DVT (OR 0.38 [0.91, 0.13], *P* = .048), or chronic obstructive pulmonary disease (OR 0.40 [0.91, 0.15], *P* = .044). After adjusting for patient variables in the multivariate analysis, patients were at significantly higher odds of SDD if they were male (OR 1.83 [2.66, 1.27], *P* = .001) or had a history of tobacco use (1.87 [3.00, 1.15], *P* = .01). They were at lower odds if they were older in age (OR 0.94 [0.96, 0.92], *P* < .001) or of higher BMI (OR 0.95 [0.99, 0.91], *P* = .006).

**Table 5:** Binary logistic regression of variables predisposing risk of same-day discharge for the post-COVID cohort.

Univariate				Multivariate			
Variable	Odds ratio	95% CI	*P*	Variable	Adjusted odds ratio	Adjusted coefficient (95% CI)	*P*
Procedure				Procedure			
Total knee arthroplasty	0.67	(0.94, 0.47)	**.02**	Total knee arthroplasty	-	-	-
Total hip arthroplasty	Ref.			Total hip arthroplasty	Reference		
Age (y)	0.94	(0.96, 0.92)	**<.001**	Age (y)	0.94	(0.96, 0.92)	**<.001**
Sex				Sex			
Male	2.15	(3.04, 1.52)	**<.001**	Male	1.83	(2.66, 1.27)	**.001**
Female	Ref.			Female	Reference		
Body mass index (kg/m^2^)	0.98	(1.01, 0.95)	.221	Body mass index (kg/m^2^)	0.95	(0.99, 0.91)	**.006**
Stroke	0.24	(0.69, 0.06)	**.02**	Stroke	-	-	-
Deep vein thrombosis	0.38	(0.91, 0.13)	**.048**	Deep vein thrombosis	0.41	(1.02, 0.13)	.078
COPD	0.40	(0.91, 0.15)	**.044**	COPD	0.41	(0.998, 0.14)	.067
Tobacco use	1.98	(3.05, 1.29)	**.002**	Tobacco use	1.87	(3.00, 1.15)	**.01**

CI, confidence interval; COPD, chronic obstructive pulmonary disease.

Bold values: statistically significant.

## Discussion

The unprecedented challenge of the COVID-19 pandemic has catalyzed changes in the efficiency dynamics of orthopaedic surgery. The stress on health-care systems required resource-sparing practices, and a shift to outpatient TJA was suggested [6]. Utilizing rapid recovery protocols for TJA has allowed for an increase in SDD and improved recovery rates without increasing adverse events [24]. These protocols work by focusing on patient factors such as patient education and support and surgical factors such as multimodal pain management, optimizing preoperative comorbidities, and early physical therapy sessions [25]. Through these techniques, we achieved equivocal rates of 30-day ED visits, readmissions, and reoperations when comparing our pre-COVID and post-COVID groups despite the rapid increase of SDD rate. This finding is supported by literature examining adverse events of revision TJA patients using rapid recovery models [11].

We found no increase in postoperative adverse outcomes between the inpatient and SDD groups, which is consistent with previous studies showing that SDD is not associated with increased adverse outcomes [11,[26], [27], [28]]. In our study, we observed post-COVID TKA patients had significantly lower prevalence of DVT history than the pre-COVID TKA patients. They also had a lower prevalence of PE history than pre-COVID TKA patients. These findings may suggest a shift in surgical candidate selection during the COVID-19 pandemic to mitigate the risks posed by prior DVT instances [29]. Previous studies have demonstrated that patients with a history of DVT who undergo lower-limb joint arthroplasty have a higher incidence of DVT during the 90-day postoperative period than those with no history of DVT [29,30].

We found male sex, lower BMI, positive tobacco use history, and lower age had higher odds of being discharged on the same day, in concordance with the literature [28,31,32]. It is important to note the risk factors for SDD in this study agree with others despite the lack of specific patient selection criteria. Furthermore, it is a significant finding of our study that these factors did not change in the midst of the COVID-19 pandemic. A significantly different finding of ours was tobacco use history, which was found to be less prevalent in SDD patients in other studies [27]. Our study did not distinguish between current and former smokers at the time of surgery; however, active nicotine use is a contraindication for elective TJA surgery at our center. We considered both former and current smokers as positive for smoking history.

Our center does not adhere to formal SDD protocol criteria for patient selection, and patients were considered for SDD largely based on their desire to be discharged, social support availability, and procedural complications. The open criteria for SDD remained the same before and after COVID; however, physical therapy sessions were optimized, with patients receiving a single required therapy session. This practice was adopted first by the THA patients, which may explain the difference seen in SDD rate between THA and TKA. Bodrogi et al. created an outpatient TJA discharge model that had absolute and relative exclusion criteria for outpatient TJA [33]. None of our SDD patients fell into the absolute exclusion criteria, but many had relative exclusion criteria, with 18.8% having diabetes mellitus, 17.2% having a history of obstructive sleep apnea, and 3.8% having a history of myocardial infarction, with inpatient and SDD patients having the same safety outcomes for these criteria. We did have SDD patients with an ASA score of 3 (30.8%) and a history of DVT (2.7%), but the numbers were statistically significantly less than the inpatient population.

With regard to the ASA score, an ASA >2 is a relative exclusion criteria for TJA SDD, with an ASA ≥3 having an OR of 2.1 for length of stay and 2.3 for any complications; however, our rate of postprocedural adverse events in the SDD group was the same as that in the inpatient group, even with 30.8% of SDD patients having ASA scores of 3, illustrating how modern rapid recovery models have advanced and allowed a greater patient population to have SDD [34]. The Outpatient Arthroplasty Risk Assessment score is widely used and combines various medical conditions and comorbidities to create a unifying score to decide if a patient is fit for SDD [35]. An Outpatient Arthroplasty Risk Assessment score <59 had a stronger positive predictive value for SDD (81.6) than ASA (56.4%), illustrating the need of a new risk assessment score other than ASA to determine the likelihood of SDD.

This study is not without limitations. This was a nonrandomized, retrospective observational study based at a single tertiary care facility. We only considered THA patients who had a DA approach and did not analyze other approaches to elective hip arthroplasty. Potentially confounding points may include the removal of THA from the IPO list; however, TKA was taken off in 2018, and we still found a considerable increase in SDD for TKA. This finding might suggest the general increase in SDD practices may be due to a response to COVID-19, with less weight placed on the removal of THA from the IPO list in January 2020. Other variables that were not considered in this study that may have an impact on SDD include MeNTS score, anesthesia type, surgical start time, and surgical duration. Additionally, the risk profile of the post-COVID cohort may have been reduced based on MeNTS score stratification, which may have contributed to selection bias. However, as these were elective total joint procedures, the demographic profile did not significantly differ between the pre-COVID and post-COVID groups overall. Further exploration of the reasons for increased smoking prevalence and SDD may also be of interest for future investigation. Our study was also limited in the follow-up duration due to the timing of the pandemic. A future study may include longer follow-up and complete pre-COVID and post-COVID cohorts defined by a current-cases threshold.

## Conclusions

At our institution, the COVID-19 pandemic was a catalyst for accelerated adoption of SDD pathways without increasing ED visits, reoperations, or readmissions. Patient factors associated with an increased likelihood of SDD did not change as a result of the pandemic. Our results support continued utilization of rapid recovery methods to reduce LOS without increasing short-term adverse events.

## Appendix A Supplementary Data

Conflict of Interest Statement for Cleary

Conflict of Interest Statement for Coobs

Conflict of Interest Statement for Martinkovich

Conflict of Interest Statement for Moskal

Conflict of Interest Statement for Samuel

Conflict of Interest Statement for Thomas

Conflict of Interest Statement for Coobs
